# Lower Digit Ratio (2D:4D) Indicative of Excess Prenatal Androgen Is Associated With Increased Sociability and Greater Social Capital

**DOI:** 10.3389/fnbeh.2019.00246

**Published:** 2019-12-05

**Authors:** Verena N. Buchholz, Christiane Mühle, Cohort Study on Substance Use Risk Factors, Gerhard Gmel, Johannes Kornhuber, Bernd Lenz

**Affiliations:** Addiction Medicine, Lausanne University Hospital CHUV, University of Lausanne, Lausanne, Switzerland; Addiction Switzerland, Lausanne, Switzerland; Centre for Addiction and Mental Health, Toronto, ON, Canada; University of the West of England, Frenchay Campus, Bristol, United Kingdom (Gerhard.Gmel@chuv.ch). La Source, School of Nursing Sciences, HES-SO University of Applied Sciences and Arts of Western Switzerland, Lausanne, Switzerland (m.mohler-kuo@ecolelasource.ch). Institut für Epidemiologie, Biostatistik und Prävention, Hirschengraben, Zurich, Switzerland (simon.foster@kjpd.uzh.ch). Addiction Medicine, Lausanne University Hospital CHUV, University of Lausanne, Lausanne, Switzerland (simon.marmet@chuv.ch). Addiction Medicine, Lausanne University Hospital CHUV, University of Lausanne, Lausanne, Switzerland (Joseph.Studer@chuv.ch).; Department of Psychiatry and Psychotherapy, Friedrich-Alexander University Erlangen-Nürnberg (FAU), Erlangen, Germany

**Keywords:** 2D:4D, digit ratio, sociability, aggression, opioid receptor, social behavior, isolation intolerance

## Abstract

Positive social interactions are crucial for human well-being. Elevated prenatal exposure to testosterone as indicated by a low second-to-fourth finger length ratio (2D:4D) relates to more aggressive/hostile behavior in men of low 2D:4D, especially in challenging situations. How much people enjoy interacting with others is determined by the personality trait sociability. Given its role in approach and avoidance behavior, sociability might also be influenced by prenatal sex hormones, but studies are inconclusive so far. Here, we investigated the association between 2D:4D and the personality trait sociability complemented by personal social capital and personal social network size, in a population-based cohort of 4998 men. Lower 2D:4D correlated significantly with higher trait sociability, bigger personal social capital, and larger personal social network size. These effects were consistent across both hands separately and their mean value. Furthermore, both factors of sociability (1) liking party and company of friends and (2) isolation intolerance, correlated significantly with the prenatal testosterone marker. The exploratory analysis revealed no link between 2D:4D and responses to the personality trait aggression items or items of anti-social-personality disorder. Our data suggest that prenatal androgen exposure organizes the brain with lasting effects on social behavior.

## Introduction

During the early prenatal window, androgens and estrogens influence the development with long-lasting effects on the structure and composition of the body and on behavior. Prenatal stress relates to increased androgen load; accordingly, intervention programs to reduce maternal stress during pregnancy are being developed (Lenz et al., 2018b). Animal models (mice, sheep) have shown permanent organizational effects of prenatal testosterone on the brain (Brown et al., 2015; Huber et al., 2018). These early effects also contribute to sex differences in adult behavior.

Aggression and social relationships are subject to gender dimorphisms. In comparison to men, women show less direct aggressive behavior (Archer, 2004), but in conduct disorder, women show hurtful manipulation of relationships (relational aggression) more often than men (Ackermann et al., 2019). Men less often report having a close confident other than the spouse (Antonucci, 1994), spend less time involved in responding to requests from others (Kessler et al., 1985; Troisi, 2001) and on online social networks (Bouna-Pyrrou et al., 2015, 2018), have a smaller risk to use them pathologically (Bouna-Pyrrou et al., 2015), and are less often the target of online communication (Griffiths et al., 2004). The sex differences may suggest that prenatal exposure to androgens influences aggressive and social behaviors in adulthood. Due to ethical reasons and the long time interval between the prenatal window and adulthood, it is hardly possible to directly investigate the effects of intrauterine sex hormones. Hence, biomarkers have been established. The second-to-fourth finger length ratio (2D:4D) is widely used to study prenatal sex hormone exposure. Reinforced prenatal androgen signaling causes lower 2D:4D in mice (Zheng and Cohn, 2011) and indirect effects of such organizational properties have been also found in humans (Manning et al., 2014), for critical review see Berenbaum et al. (2009), Del Giudice et al. (2018). E.g., human maternal plasma testosterone during pregnancy shows a negative correlation with new borns’ digit ratio in both sexes (Ventura et al., 2013), amniotic fluid testosterone is negatively related to 2 year olds’ 2D:4D (Lutchmaya et al., 2004), and females with exposure to excessive prenatal testosterone levels due to congenital adrenal hyperplasia (CAH) have lower 2D:4D values than normal controls (Brown et al., 2002; Buck et al., 2003). Hence, lower 2D:4D is indicative of higher prenatal androgen load in humans.

A meta-analysis reported that lower 2D:4D relates to more aggression in men (Hönekopp and Schuster, 2010), although these effects have been found to be small. Furthermore, situational factors, and adult hormone levels play a moderating role (Hönekopp and Watson, 2011). From an evolutionary perspective, one could also expect that social behaviors involving approach and bonding might be related to biological factors such as prenatal sex hormone exposure. Studies investigating social behavior and prenatal testosterone exposure have been conducted under varying contexts and with the use of different methods, ranging from economic games to observation of interactions. The findings have been inconsistent, perhaps due to the complexity of human behavior and its interplay with environmental factors (Millet and Buehler, 2017). Indeed, contextual factors such as the presence of aggressive (Kilduff et al., 2013) or sexual cues (Van den Bergh and Dewitte, 2006), adult hormone levels (Millet and Dewitte, 2008; van Honk et al., 2012; Manning et al., 2014; Portnoy et al., 2015; Millet and Buehler, 2017), cognitive reflection (Millet and Aydinli, 2019), and time-pressure (Bird et al., 2019) moderate the relationship between 2D:4D and prosocial behavior in economic games. Furthermore, the relationships might differ across sex (Hönekopp and Watson, 2011). However, the evidence seems to be more consistent at least at the level of achievements in adults. Within men, more prenatal androgen (lower 2D:4D) is associated with higher academic grade (Nye et al., 2017; Tektas et al., 2019), larger reproductive success (Manning et al., 2000), and higher trading outcome in financial traders (Coates et al., 2009). Thus, in contrast to what one might expect due to the above reported sex differences, men with lower (more masculine) 2D:4D perform better in tasks that require networking or bonding. Accordingly, in men, lower 2D:4D has been related to more fairness (Millet and Dewitte, 2006), stronger cognitive reflection (Bosch-Domenèch et al., 2014), and higher betweenness centrality, i.e., they connect separated parts of the social structure (Kovářík et al., 2017). Moreover, males with lower 2D:4D show more courtship behavior in social interactions with women (Roney and Maestripieri, 2004).

How much people enjoy interacting with others or need to be in company (two factors of sociability) and how many people they know to rely on (social capital) are important determinants of human well-being and health. For example, a low social capital has been associated with negative health outcomes (Murayama et al., 2012) including depression, pain, and psychosomatic symptoms (Åslund et al., 2010). Thus, associations between 2D:4D and health further highlight the importance to understand the role of prenatal androgen exposure in adult social behavior. For example, in males, lower 2D:4D has been associated with lower anxiety (Evardone and Alexander, 2009), a higher risk for conduct problems during childhood (Eichler et al., 2018), addictive and substance use disorders (Kornhuber et al., 2011, 2013; Canan et al., 2017, 2019; Lenz et al., 2017, 2018a, 2019a; Siegmann et al., 2019), suicide (Lenz et al., 2016, 2019b), and reduced life expectancy in adulthood (Lenz and Kornhuber, 2018).

Given the complexity of behavior in experimental tasks or hypothetical trading situations, relatively stable indicators of social behavior, like the personality trait sociability, personal social capital, and personal social network size provide a suitable approach to investigate the link between social behavior and organizational effects of prenatal androgens. Furthermore, the Alternative Five Model (measuring sociability as one of five factors) has been established for traits with a strong biological-evolutionary basis and increases the comparability of our results with animal models (Zuckerman et al., 1993).

Here, we tested whether 2D:4D relates to sociability, personal social capital, and personal social network size in a large population based cohort of 4998 young males. We also explored whether 2D:4D is associated with aggression and anti-social personality characteristics.

## Materials and Methods

### Study Sample

The data analyzed here originate in the third survey wave of the longitudinal Cohort Study on Substance Use Risk Factors (C-SURF)^1^. From 2010 to 2012, 7556 young males, who attended their mandatory recruitment for the Swiss army, gave written informed consent and 5987 participated in the first wave. Data for this study were derived from the third wave which has been conducted between April 2016 and March 2018 and which has included 5516 males (see^2^ for Questionnaire No. 3).

### Behavioral Phenotyping

To measure *sociability*, we used the subscale sociability of the Alternative Five Factor Model (Zuckerman-Kuhlman Personality Questionaire, ZKPQ-50-cc) (Zuckerman et al., 1993) questionnaire, consisting of 10 binary items and its summation score (Aluja et al., 2006). The scale was further divided in the two subscales representing (1) liking lively parties and friends and (2) intolerance of social isolation. *Personal social capital with the subscales bridging and bonding* was quantified by an adaptation of the Personal Social Capital Scale (Archuleta and Miller, 2011; Chang and Zhu, 2012; Wang et al., 2014) with only the 5 most relevant items per subscale selected in C-SURF and a Likert Scale 1–5 to respond. Bonding social capital refers to how well a person is embedded within their various networks of different types of people (e.g., family members, friends, and former colleagues), and bridging social capital refers to how well a person is embedded within different types of social organizations. *Personal social network size* was estimated in C-SURF by two items referring to social network size from the Personal Social Capital Scale (Archuleta and Miller, 2011; Wang et al., 2014). The first item refers to perceived number of friends (from the bonding capital subscale) and the second to the perceived number of cultural, recreational, and leisure groups/organizations in the subject’s community (from the bridging capital subscale).

*Aggression* was quantified using the 10 items scale of the ZKPQ-50-cc (Aluja et al., 2006). The score on the *Anti-Social Personality Disorder* scale was probed with items from the Mini-International Neuropsychiatric Interview (M.I.N.I.) with ASSIST-WHO (Sheehan et al., 1998; Hergueta et al., 2015).

### 2D:4D

The participants were instructed to document the lengths of their second and fourth fingers in millimeters separately for their right and left hands (see^2^, Questionnaire No. 3 ID: J18) similar to the methods described by Reimers (2007) and Lenz et al. (2018a). The instruction was “Hold your left hand in front of you. Look at where your index finger joins the palm of your hand. Find the bottom crease. Go to the middle of this crease. Put the 0 of your ruler exactly on the middle of the bottom crease (see 2a in the picture below). Make sure the ruler runs straight up the middle of your finger. Measure to the tip of your finger (not your nail see 2b in the picture) in millimeters.” Finger lengths under 10 mm or over 100 mm (Reimers, 2007) and, additionally, 2D:4D values outside of the 2.5 and 97.5 percentiles (Hell and Päßler, 2011; Lenz et al., 2018a) separately for the right and left hand were excluded. Subsequent, we calculated the mean of right-hand 2D:4D and left-hand 2D:4D (M2D:4D) which served as our primary predictor. Whereas some studies report that target traits are more strongly related to 2D:4D of the right hand (Manning et al., 1998; Hönekopp and Watson, 2010; Kornhuber et al., 2011; Masuya et al., 2015; Bilgic et al., 2016), other report stronger associations with 2D:4D of the left hand (Muller et al., 2012; Kornhuber et al., 2013; Hong et al., 2014; Lenz et al., 2017, 2019a). As far as we know, there is no reliable explanation for different associations of right- and left-hand 2D:4D with prenatal androgen load. There is also no support for superiority of either side in a meta-analysis on aggression (Hönekopp and Watson, 2011). Separate values for right-hand 2D:4D (R2D:4D), left-hand 2D:4D (L2D:4D), and the difference between R2D:4D and L2D:4D (2D:4Dr-l) were defined as exploratory predictors. Moreover, regarding quality control, we refer to a previous analysis of the same cohort (except for 9 patients with missing data on alcohol-related questions) which showed median values of 2D:4D similar to other studies (Lenz et al., 2019a).

### Statistical Analyses

Continuous data are presented as the median and interquartile range (IQR) and nominal data as frequencies (FREQUENCIES function in SPSS). For missing data points, the corresponding study subjects were excluded from the specific analyses and the number of individuals included in these analyses is reported. Correlations were calculated using Spearman’s method, because normal distribution was rejected for all variables. We used the Mann–Whitney *U* test to compare independent groups. For two-sided tests, *p* < 0.05 was considered to be statistically significant. All reported *p*-values are uncorrected for multiple comparisons. Data were analyzed using IBM SPSS Statistics Version 21 for Windows (SPSS Inc., Chicago, IL, United States) and Graph Pad Prism 5 (Graph Pad Software Inc., San Diego, CA, United States).

## Results

### Sample Characteristics

Due to missing values or eliminations resulting from quality control of R2D:4D and L2D:4D, 518 individuals were excluded from the statistical analyses. This resulted in a total cohort of 4998 study subjects and M2D:4D, L2D:4D, and R2D:4D sub-cohorts of 4778, 4898, and 4878 individuals. The total cohort was characterized as follows: age 25 years (IQR 25–26; *N* = 4998); body mass index 23.5 kg/m^2^ (IQR 21.8–25.5; *N* = 4990); 79.6% gainfully employed (*N* = 4997); 3.0% secondary education, 1.2% basic vocational education, 34.4% secondary vocational/technical education, 4.3% community college, 11.2% vocational high school, 11.8% high school, 23.4% bachelor (university), 6.1% master (university), 4.6% other (*N* = 4985); 82.9% single, 5.2% married, 0.1% divorced, 11.6% not married, not separated, not divorced but living together with my partner (e.g., in registered partnership), 0.1% married but separated, 0.1% widowed (*N* = 4989).

### Trait Sociability

Lower M2D:4D (indicative of higher levels of prenatal androgen exposure) correlated with higher trait sociability (ρ = −0.043, *N* = 4755, *p* = 0.003), and both L2D:4D and R2D:4D correlated similarly with trait sociability (ρ = −0.045, *N* = 4875, *p* = 0.002; ρ = −0.032, *N* = 4855, *p* = 0.024). 2D:4Dr-l did not correlate with trait sociability (*p* > 0.05). As shown in Table 1, both subscales of sociability correlated significantly with 2D:4D.

**TABLE 1:** *Post hoc* analysis Sociability: Spearman correlations at facet level.

**Sociability**		**M2D:4D**	**L2D:4D**	**R2D:4D**	**2D:4Dr-l**
Parties/Friends	ρ	–0.036	–0.031	–0.034	–0.008
	*p*	**0.012**	**0.029**	**0.019**	0.600
	*N*	4763	4883	4863	4763
Isolation Intolerance	ρ	–0.035	–0.041	–0.020	0.018
	*p*	**0.017**	**0.005**	0.172	0.221
	*N*	4760	4880	4860	4760

*2D:4D, second-to-fourth-finger length ratio; primary predictor: M2D:4D, mean of R2D:4D and L2D:4D; exploratory predictors: L2D:4D, left-hand 2D:4D; R2D:4D, right-hand 2D:4D; 2D:4Dr-l, difference between R2D:4D and L2D:4D. *P* < 0.05 (uncorrected) in bold. Cronbach’s alpha: Parties/Friends 0.48, Isolation Intolerance 0.57.*

Statistics for the M2D:4D differences for the 10 individual binary items (*post hoc* analysis) are shown in Supplementary Table S1. Specifically, the items “At parties, I enjoy mingling with many people whether I already know them or not.” and “I am a very sociable person.” were significantly associated with lower M2D:4D.

### Personal Social Capital

Lower M2D:4D correlated with bigger personal social capital (ρ = −0.040, *N* = 5762, *p* = 0.005), and both L2D:4D and R2D:4D correlated similarly with bigger personal social capital (ρ = −0.036, *N* = 4882, *p* = 0.012; ρ = −0.013, *N* = 4861, *p* = 0.039). 2D:4Dr-l did not correlate with personal social capital (*p* > 0.05). Table 2 shows the results of the *post hoc* analysis on subscale level.

**TABLE 2:** *Post hoc* analysis Personal social capital: Spearman correlations at subscale level.

**Personal social capital**		**M2D:4D**	**L2D:4D**	**R2D:4D**	**2D:4Dr-l**
	ρ	−0.032	−0.032	−0.030	0.011
Bridging	*p*	**0**.**026**	**0**.**026**	**0**.**035**	0.443
	*N*	4764	4884	4863	4764

	ρ	−0.038	−0.031	−0.032	–0.001
Bonding	*p*	**0**.**009**	**0**.**030**	**0**.**025**	0.968
	*N*	4768	4888	4867	4768

*2D:4D, second-to-fourth-finger length ratio; primary predictor: M2D:4D, mean of R2D:4D and L2D:4D; exploratory predictors: L2D:4D, left-hand 2D:4D; R2D:4D, right-hand 2D:4D; 2D:4Dr-l, difference between R2D:4D and L2D:4D. *P* < 0.05 (uncorrected) in bold. Cronbach’s alpha: Bridging 0.79, Bonding 0.83.*

Item level analysis revealed significant correlations with the items “interacting with people makes me feel like a part of a large community,” “the people I interact with would be good job references for me” and “if I needed an emergency loan, I know someone I can turn to”, for details see Table 3.

**TABLE 3:** *Post hoc* analysis Personal social capital: Spearman correlations at item level.

**Personal social capital**	**M2D:4D**			**L2D:4D**			**R2D:4D**			**2D:4Dr-l**		
				
	**ρ**	***p***	***N***	**ρ**	***p***	***N***	**ρ**	***p***	***N***	**ρ**	***p***	***N***
Interacting with people makes me want to try new things	–0.026	0.077	4771	–0.021	0.138	4891	–0.023	0.107	4870	0.009	0.514	4771
Interacting with people makes me interested in what people unlike me are thinking	–0.018	0.206	4771	–0.019	0.189	4891	–0.013	0.355	4870	0.009	0.535	4771
Interacting with people makes me feel like a part of a large community	–0.038	**0**.**009**	4770	–0.034	**0**.**019**	4890	–0.036	**0**.**011**	4869	0.009	0.551	4770
Interacting with people makes me feel connected to the bigger picture	–0.008	0.567	4765	–0.014	0.322	4885	–0.010	0.492	4864	0.016	0.281	4765
I come into contact with people all the time	–0.012	0.390	4770	–0.016	0.258	4890	–0.006	0.693	4869	0.012	0.400	4770
There are several people I trust to solve my problems	–0.024	0.099	4769	–0.026	0.074	4889	0.013	0.380	4868	0.013	0.365	4769
If I needed an emergency loan, I know someone I can turn to	–0.037	**0**.**010**	4769	–0.027	0.061	4889	–0.033	**0**.**022**	4868	–0.006	0.700	4769
There is someone I can turn to for advice about making very important decisions	–0.016	0.261	4770	–0.008	0.589	4890	–0.020	0.170	4869	–0.019	0.193	4770
I know several people well enough to get them to do anything important	–0.020	0.165	4770	–0.017	0.224	4890	–0.017	0.235	4869	0.001	0.963	4770
The people I interact with would be good job references for me	–0.031	**0**.**031**	4770	–0.027	0.057	4890	–0.025	0.082	4869	0.010	0.503	4770

*2D:4D, second-to-fourth-finger length ratio; primary predictor: M2D:4D, mean of R2D:4D and L2D:4D; exploratory predictors: L2D:4D, left-hand 2D:4D; R2D:4D, right-hand 2D:4D; 2D:4Dr-l, difference between R2D:4D and L2D:4D. *P* < 0.05 (uncorrected) in bold.*

### Personal Social Network Size

**FIGURE 1:**
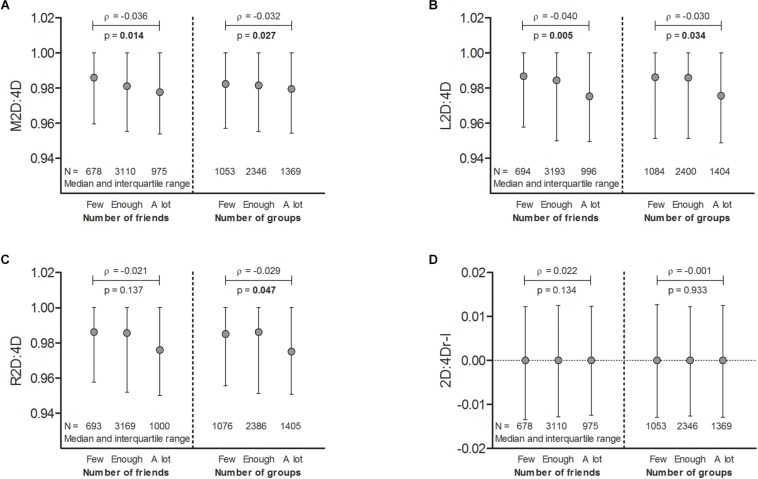
The M2D:4D **(A)**, L2D:4D **(B)**, and R2D:4D **(C)** ratios [but not 2D:4Dr-l **(D)**] were negatively correlated with self-reports for the number of friends and the number of cultural, recreational, and leisure groups/organizations/associations in one’s community. 2D:4D, second-to-fourth-finger length ratio; primary predictor: M2D:4D, mean of R2D:4D and L2D:4D; exploratory predictors: L2D:4D, left-hand 2D:4D; R2D:4D, right-hand 2D:4D; 2D:4Dr-l, difference between R2D:4D and L2D:4D. *P* < 0.05 (uncorrected) in bold.

2D:4D correlated negatively with the personal social network size (Figure 1).

### Aggression and Anti-social Personality

M2D:4D, L2D:4D, R2D:4D, or 2D:4Dr-l did not correlate with aggression or anti-social personality disorder score (*p* > 0.05, Supplementary Table S2).

## Discussion

Here, we report that higher sociability and bigger personal social capital are correlated with lower 2D:4D in a population-based cohort of young Swiss men. Notably, both factors of sociability, liking lively parties and friends and intolerance of social isolation (Zuckerman et al., 1993), correlated independently with 2D:4D across both hands. Furthermore, we provide preliminary evidence for an association between bigger personal social network size and lower 2D:4D. These results suggest that, in men, higher prenatal androgen exposure improves sociability and leads to a bigger social capital and social network size in adulthood. Our observation is consistent with a study showing that prenatal testosterone as measured in amniotic fluid during 13–20 weeks of gestation is associated with approach behavior and reactivity to happy faces in brain reward areas of boys (Lombardo et al., 2012). The large sample size of nearly 5000 study participants analyzed here is a major strength of this project. It is limited by the 2D:4D self-measurement method which is related to reduced reliability in comparison to expert measured 2D:4D (Hönekopp and Watson, 2010).

Sociability involves the opioid system of the brain (Knowles et al., 1989; Kalin et al., 1995). In animal experiments, prenatal androgen receptor inhibition by flutamide down-regulates cerebral expression of the μ opioid receptor 1 in adulthood (Huber et al., 2018). In line with this association between prenatal sex hormone effects and opioid signaling, R2D:4D in men has been related to genetic polymorphisms in opioid receptors (Pearce et al., 2018). During social laughter – related to the sociability factor “party and friends” – endogenous opioids are released, and the depletion during social isolation motivates to seek company – related to the sociability factor “isolation intolerance” (Knowles et al., 1989; Kalin et al., 1995). The minor G-allele of the μ-opioid receptor 1 polymorphism rs1799971 is associated with more pleasure experienced in social situations (Troisi et al., 2011), and mice with this variant have increased motivation for non-aggressive social interactions and show less avoidance after social defeat (Briand et al., 2015). Taken together, prenatal androgen exposure may organize cerebral opioid signaling with behavioral effects on sociability. Future research should investigate how prenatal influences might interact with genetics to affect sociability.

We found lower 2D:4D to be associated with higher sociability. Our findings are in line with previous reports on higher betweenness centrality in men with lower 2D:4D, i.e., these subjects connect separated parts of the social structure (Kovářík et al., 2017). Furthermore, academic, reproductive, and trading success, all negatively correlated with 2D:4D (Manning et al., 2000; Coates et al., 2009; Nye et al., 2017; Tektas et al., 2019), have networking as an essential common mechanism to success. Hence, higher sociability might mediate the relationship between lower 2D:4D and successfulness in men.

In our adult cohort, we did not find any significant correlation between 2D:4D and aggression, which might be explained by the low precision due to the employed self-measurement technique and the fact that correlations of aggression and 2D:4D in adults are mainly found in challenging situations (Hönekopp and Watson, 2011) and in other situations are small at the best (Hönekopp and Watson, 2011).

By contrast, we found lower 2D:4D to be associated with higher sociability. At the first glance, our findings may contradict that lower 2D:4D (indicative of higher prenatal androgen exposure) relates to behavioral symptoms in boys (Williams et al., 2003; Eichler et al., 2018), which entails problems in social interaction. Aggression, fighting, and lacking obedience are characteristics of conduct disorder. However, the frontal lobe and cognitive reflection are still developing in children. Frontal lobe development and cognitive reflection inhibit aggressive outbursts and the shift of neural regulation to prefrontal areas takes place during puberty (Cubillo et al., 2012; Rubia et al., 2013; Tyborowska et al., 2016). In adulthood, cognitive reflection is higher in individuals with lower 2D:4D (Bosch-Domenèch et al., 2014) and probably explains the moderating role of sexual and aggressive cues on the relationship between 2D:4D and aggressive behavior (Hönekopp and Watson, 2011). Without a situationally triggered testosterone surge, aggression as a trait is less evident in daily life and cognitive reflection might counteract aggressive trends in men with low 2D:4D. Boys with higher sociability (following higher prenatal androgen load) may be involved into fights more often due to the increased total frequency of interactions with others and given the fact that physical aggression is used instrumentally in healthy young children.

In support of this developmental view on aggression, we also did not find a correlation of prenatal testosterone with anti-social personality disorder (ASPD) items. Whereas conduct disorder increases the risk for ASPD (Olsson and Hansson, 2009), other factors like intelligence, parent psychopathology, parent-child relation, and peer-rejection are known to moderate this risk essentially (Olsson and Hansson, 2009).

In this study, lower 2D:4D correlated with bigger personal social capital and a larger personal social network. Here, we will argue that negotiation strategies, which are conceptually related to social networking, change from children to adulthood into more functional behavior in people with lower 2D:4D. In adult men, lower 2D:4D is associated with more uncooperative behavior, but only when they act intuitively or less reflected (Millet and Buehler, 2017; Millet and Aydinli, 2019) and as already mentioned, men with lower 2D:4D have stronger cognitive reflection skills (Bosch-Domenèch et al., 2014). In general, adult men with low 2D:4D prefer fair from either altruistic or egoistic choices (Millet and Dewitte, 2006), even though their faces appear more dominant to others (Neave et al., 2003). In children, however, a lower 2D:4D is still unrelated to fair choices and correlates with less altruistic choices instead (Millet and Dewitte, 2006). In adults, social status relevance (potentially leading to a surge in testosterone) within a given context moderates the impact of 2D:4D on cooperative behavior, aggression, and dominance in economic games (Millet and Buehler, 2017). Taken together, evidence on negotiation strategies of lower 2D:4D subjects supports our findings on the relationship between 2D:4D, social capital, and network size.

Furthermore, children with a higher status – as measured in number of friends/interaction partners – choose the prosocial option less often (Horn et al., 2018). In contrast to our data from adults, in which a bigger social capital and a larger social network are associated with lower 2D:4D, in boys the strategies to gain status may still be dysfunctional, as a link with number of friends/interaction partners and 2D:4D was not found (Horn et al., 2018).

The relationship between sociability, aggression, and behavioral strategies to gain status or bond might change from childhood to adulthood, when cognitive reflection and the frontal lobe have fully developed. As a consequence, normative behavior, learned cooperation, and fairness may be utilized by adult men with low 2D:4D, at least in unchallenging situations. Furthermore, experiences from frequent social interactions (sociability) and from testing the limits with others during childhood (instrumental aggression) might in the end help to bond with others and make these subjects more resilient, explaining the long term positive outcomes of men with lower 2D:4D in academia (Nye et al., 2017; Tektas et al., 2019), reproduction (Manning et al., 2000) and trading (Coates et al., 2009).

Although we found that low 2D:4D in men is associated with higher trait sociability and possibly more social bonds to rely on, there is evidence for a more avoidant attachment style (Del Giudice and Angeleri, 2016) and lesser quality of relationships in people with low 2D:4D (Knickmeyer et al., 2005). Furthermore, intimate partner violence is actually higher in low 2D:4D men (Romero-Martínez et al., 2013). Thus, sociability and a bigger social capital in men do not necessarily mean that intimate or close relationships are better on the long term. They might even be worse as subjects are more directed at social status than intimacy.

G-allele carriers of the μ opioid receptor 1 polymorphism rs1799971 experience more pleasure in social situations (Troisi et al., 2011) and alcohol-dependent G-allele carriers show increased cue-reactivity to alcohol stimuli in certain brain regions which correlates with craving (Bach et al., 2015). As endogenous opioids contribute to the punishing effects of social isolation and rejection (Knowles et al., 1989; Kalin et al., 1995; Briand et al., 2015), it is interesting that an interaction between 2D:4D and the rs1799971 polymorphism has been reported for alcohol dependence (Gegenhuber et al., 2018). Both aspects of sociability, the interest in parties and friends und isolation intolerance, which correlated with 2D:4D in our study, might influence the development of alcohol dependency. This study’s results indicate that the pleasure to bond with others and enjoy social laughter is increased in people with low 2D:4D which might lead to more reward (opioid release) experienced during these situations. This mechanism might potentiate the rewarding effect of consumption (again opioid release) by increased chances of social laughter and bonding. Finally, also isolation intolerance might play a role, as it might induce drinking behavior to cope with loneliness. However, further research is needed to test these hypotheses.

At first glance, the observed negative correlation between 2D:4D and social network might contradict the fact that lower 2D:4D has been associated with suicide completion (Lenz et al., 2016) because social connectedness has been shown to be protective against suicidal behaviors (Fässberg et al., 2012). However, for suicide completion, it has been argued that correlations of lower 2D:4D with stronger cognitive reflection (Bosch-Domenèch et al., 2014; Millet and Aydinli, 2019) might play a role, leading to better planned and more successful suicide attempts, as 2D:4D measured independently from cognitive reflection is unrelated to suicidal thoughts and attempts (Lenz et al., 2019b).

## Limitations

Self-measured 2D:4D is less reliable than expert-measures and is said to reach only 46% of its reliability (Hönekopp and Watson, 2010). Furthermore, finger deformation was not assessed in this project, which has reduced precision. We are aware of current criticism on 2D:4D as a proxy for prenatal androgen exposure, as the experimental evidence used to support the validity of 2D:4D as a biomarker of prenatal androgen exposure has not been replicated consistently (Berenbaum et al., 2009; Huber et al., 2017; Del Giudice et al., 2018).

Besides correlating our primary independent variable with our dependent variables, we extended the analysis to exploratory testing of left and right hand 2D:4D and asymmetries of left and right hand 2D:4D, but did not correct for multiple hypothesis testing which might have resulted in false positive findings in the exploratory analysis.

Personal social capital was assessed in C-SURF only with a selection of items from the Personal Social Capital Scale, using only the 5 most relevant items per subscale. Even though we found a good internal consistency of 0.85 Cronbach’s alpha, construct validity remains unknown for this subset of items.

Personality disorder diagnoses like anti-social personality disorder should be assessed by experienced clinicians using structured clinical interviews (Paap et al., 2017). Here, we correlated the summation score of a self-report screening instrument with unknown discriminability for this clinical disorder.

We did not find a correlation between 2D:4D and aggression as a personality factor. In a meta-analysis on 2D:4D and aggressive behavior, it was reported that any correlation found appear to be very small and findings are context dependent (Hönekopp and Watson, 2011). We investigated the personality factor aggression with a questionnaire and did not use an experimental setup with provocative cues or interaction partners. Furthermore, we face a lower reliability of self-measured 2D:4D measures in comparison to expert ratings. Moreover, sex differences in aggression appear to be larger in children than in adults (Campbell, 2006; Archer, 2009) and our adult cohort is rather homogeneous in age.

Exploratory analysis of social network size was only probed by two self-reported items and future research should use more reliable and objective measures to investigate the relationship between 2D:4D and social network size.

Finally, our cohort consisted of mostly Caucasian young men and the results cannot be transferred to other ethnicities, gender, or age groups.

## Conclusion

To summarize, our data show that low 2D:4D is associated with higher trait sociability, bigger personal social capital, and larger personal social network size. Given the complexity of human behavior and environmental/nurture effects on personality, it is not surprising that the correlations are small though. Our study provides a better understanding of the link between prenatal influences and social behavior in adulthood. It also leads to an interesting hypothesis on the mediating role of sociability between prenatal environment and life achievements, behavioral problems in adolescence, and other health related aspects.

## Members of Cohort Study on Substance Use Risk Factors

Gerhard Gmel: Addiction Medicine, Lausanne University Hospital CHUV, University of Lausanne, Lausanne, Switzerland; Addiction Switzerland, Lausanne, Switzerland; Centre for Addiction and Mental Health, Toronto, ON, Canada; University of the West of England, Frenchay Campus, Bristol, United Kingdom (Gerhard.Gmel@chuv.ch). Meichun Mohler-Kuo: La Source, School of Nursing Sciences, HES-SO University of Applied Sciences and Arts of Western Switzerland, Lausanne, Switzerland (m.mohler-kuo@ecolelasource.ch). Simon Foster: Institut für Epidemiologie, Biostatistik und Prävention, Hirschengraben, Zurich, Switzerland (simon.foster@kjpd.uzh.ch). Simon Marmet: Addiction Medicine, Lausanne University Hospital CHUV, University of Lausanne, Lausanne, Switzerland (simon.marmet@chuv.ch). Joseph Studer: Addiction Medicine, Lausanne University Hospital CHUV, University of Lausanne, Lausanne, Switzerland (Joseph.Studer@chuv.ch).

## Data Availability Statement

The datasets generated for this study are available on request to the corresponding author.

## Ethics Statement

This study was approved by the Ethics Committee for Clinical Research of Lausanne University Medical School (Protocol No. 15/07). The patients/participants provided their written informed consent to participate in this study.

## Author Contributions

VB and BL conceived and designed the research, analyzed the data, and wrote the manuscript. GG, MM-K, SM, SF, and JS performed the experiments. CM and JK commented on the manuscript and provided the intellectual input.

## Conflict of Interest

The authors declare that the research was conducted in the absence of any commercial or financial relationships that could be construed as a potential conflict of interest.

## Footnotes

1 www.c-surf.ch

2 www.c-surf.ch/img/questionnaires_pdf/q3_follow_up2_en.pdf
