# Supplementary material for: Lower Digit Ratio (2D:4D) Indicative of Excess Prenatal Androgen Is Associated With Increased Sociability and Greater Social Capital
Source: Front Behav Neurosci. 2019 Dec 5;13:246. doi: 10.3389/fnbeh.2019.00246 (PMC6906175; doi:10.3389/fnbeh.2019.00246)
Supplement: Supplementary file 1 [file Table_1.docx]

**Supplementary Table 1: M2D:4D compared for each binominal response**

|  |  | M2D:4D |  |  | L2D:4D |  |  | R2D:4D |  |  | 2D:4Dr-l |  |
| --- | --- | --- | --- | --- | --- | --- | --- | --- | --- | --- | --- | --- |
| **Sociability items** | N | U | p | N | U | p | N | U | p | N | U | p |
| *I do not mind going out alone and usually prefer it to being out in a large group* | 4772 | 1,862,242 | 0.091 | 4892 | 1,985,927 | 0.168 | 4872 | 1,967,248 | 0.212 | 4772 | 1,927,831 | 0.986 |
| *I spend as much time with my friends as I can* | 4770 | 2,723,053 | 0.111 | 4890 | 2,846,757 | 0.051 | 4870 | 2,848,254 | 0.161 | 4770 | 2,776,276 | 0.637 |
| I do not need a large number of casual friends | 4772 | 2,081,007 | 0.644 | 4892 | 2,185,191 | 0.479 | 4872 | 2,174,998 | 0.643 | 4772 | 2,097,692 | 0.957 |
| I tend to be uncomfortable at big parties | 4772 | 2,323,548 | 0.272 | 4892 | 2,449,884 | 0.321 | 4872 | 2,441,507 | 0.467 | 4772 | 2,359,358 | 0.783 |
| At parties, I enjoy mingling with many people whether I already know them or not | 4773 | 2,695,220 | **0.014** | 4893 | 2,859,906 | 0.055 | 4873 | 2,814,554 | **0.015** | 4773 | 2,751,558 | 0.198 |
| *I would not mind being socially isolated in some place for some period of time* | 4773 | 2,753,899 | 0.105 | 4893 | 2,885,712 | 0.064 | 4873 | 2,917,476 | 0.496 | 4773 | 2,788,213 | 0.362 |
| *I like to be alone so I can do things I want to do without social distractions* | 4769 | 2,746,665 | 0.077 | 4889 | 2,862,016 | 0.022 | 4869 | 2,920,908 | 0.557 | 4769 | 2,742,654 | 0.061 |
| I am a very sociable person | 4771 | 2,207,327 | **0.034** | 4891 | 2,344,358 | 0.086 | 4871 | 2,300,607 | **0.032** | 4771 | 2,294,946 | 0.947 |
| *I usually prefer to do things alone* | 4772 | 2,748,285 | 0.287 | 4892 | 2,858,796 | 0.088 | 4872 | 2,897,600 | 0.697 | 4772 | 2,761,509 | 0.427 |
| I probably spend more time than I should socializing with friends | 4771 | 941,359 | 0.188 | 4891 | 1,003,978 | 0.203 | 4871 | 1,005,502 | 0.354 | 4771 | 938,794 | 0.154 |

P < 0.05 (uncorrected for multiple hypothesis testing) in bold; Italics: Facet Isolation Intolerance

| **Supplementary Table 2: Spearman correlations of 2D:4D with Anti-Social Personality Disorder score and Aggression/Hostility** | | | | | |
| --- | --- | --- | --- | --- | --- |
|  | | M2D:4D | L2D:4D | R2D:4D | 2D:4Dr-l |
| Anti-Social Personality Disorder score | ρ | -0.002 | 0.001 | -0.002 | -0.014 |
|  | p | 0.916 | 0.928 | 0.913 | 0.324 |
|  | N | 4766 | 4886 | 4866 | 4766 |
| Aggression/ Hostility | ρ | 0.007 | -0.005 | 0.019 | 0.024 |
|  | p | 0.631 | 0.747 | 0.179 | 0.099 |
|  | N | 4760 | 4880 | 4860 | 4760 |
